# Data on diagnostic performance of stress perfusion cardiac magnetic resonance for coronary artery disease detection at the vessel level

**DOI:** 10.1016/j.dib.2017.11.096

**Published:** 2017-12-07

**Authors:** Apostolos Kiaos, Ioannis Tziatzios, Stavros Hadjimiltiades, Charalambos Karvounis, Theodoros D. Karamitsos

**Affiliations:** 1st Department of Cardiology, Aristotle University of Thessaloniki, Thessaloniki, Greece

**Keywords:** Cardiovascular magnetic resonance, Stress perfusion, Coronary artery disease, Diagnostic accuracy meta-analysis

## Abstract

Stress perfusion cardiac magnetic resonance (CMR) has been proposed as an important gatekeeper for invasive coronary angiography (ICA) and percutaneous coronary interventions (PCI) in patients evaluated for possible coronary artery disease (CAD) (Fihn et al., 2012; Montalescot et al., 2013) [1], [2]. Several meta-analyses have evaluated the accuracy of stress perfusion CMR to diagnose CAD at the vessel level (Danad et al., 2017; Dai et al., 2016; Jiang et al., 2016; Takx et al., 2015; Li et al., 2015; Desai and Jha, 2013; Jaarsma et al. 2012; Hamon et al., 2010; Nandalur et al. 2007) [3], [4], [5], [6], [7], [8], [9], [10], [11]. However, they included in the same analysis studies with different definitions of significant CAD (i.e. fractional flow reserve [FFR] < 0.75 and < 0.80 or coronary stenosis ≥ 50% and ≥ 70%), magnetic field strength (1.5 or 3 Tesla [T]), and study protocol (integration or not of late gadolinium enhancement [LGE] into stress perfusion protocol). Data of 34 studies (6091 arteries) have been pooled with the aim of analyzing the accuracy of stress perfusion CMR for the diagnosis of ischemic heart disease at the vessel level according to different definitions of significant CAD, magnetic field strength and study protocol (Arnold et al., 2010; Bettencourt et al., 2013; Cheng et al., 2007; Chiribiri et al., 2013; Cury et al., 2006; De Mello et al., 2012; Donati et al., 2010; Ebersberger et al., 2013; Gebker et al., 2008; Greulich et al., 2015; Hussain et al., 2016; Ishida et al., 2005, 2003; Kamiya et al., 2014; Kitagawa et al., 2008; Klein et al., 2008; Klem et al., 2006; Klumpp et al., 2010; Krittayaphong et al., 2009; Lockie et al., 2011; Ma et al., 2012; Merkle et al., 2007; Meyer et al., 2008; Mor-Avi et al., 2008; Pan et al., 2015; Papanastasiou et al., 2016; Pons Lladó et al., 2004; Sakuma et al., 2005; Salerno et al., 2014; Scheffel et al., 2010; van Werkhoven et al., 2010; Walcher et al., 2013; Watkins et al., 2009; Yun et al., 2015) [12–45]. This article describes data related article titled “Diagnostic Performance of Stress Perfusion Cardiac Magnetic Resonance for the Detection of Coronary Artery Disease” (Kiaos et al., submitted for publication) [46].

**Specifications Table**

**Table:** 

Subject area	Medicine; Meta-analysis
More specific subject area	Cardiology; Stress perfusion cardiac magnetic resonance
Type of data	Tables; Figure
How data was acquired	Meta-analysis
Data format	Analyzed
Experimental factors	Subgroup analyses based on different definitions of significant CAD, magnetic field strength, and study protocol at the vessel level
Experimental features	34 studies evaluated the accuracy of qualitative stress perfusion CMR to diagnose significant CAD at the vessel level, of which 9 used FFR as the reference standard. Studies that were performed at 1.5-T for detecting coronary stenosis ≥ 50% and ≥ 70% were 11 and 13 respectively, and studies that were performed at 3-T for detecting coronary stenosis ≥ 50% and ≥ 70% were 5 and 4 respectively.
Data source location	UK, USA, Portugal, Brazil, Switzerland, Germany, Japan, Thailand, China, Spain, Netherlands, Taiwan
Data accessibility	Data is with this article

**Value of the data**

• Among studies performed at 1.5-T, those with FFR as the reference standard had greater diagnostic accuracy at the vessel level compared to studies using ICA.
• Integration of LGE into stress perfusion CMR protocol did not influence the diagnostic accuracy for CAD detection at the vessel level.
• Among studies using FFR as the reference standard there was no difference between 1.5 and 3-T at the vessel level in contrast to studies using anatomical reference standard where 3-T demonstrated higher diagnostic performance with a notable difference in sensitivity.
• For the 7 studies reporting data for the detection of ≥ 50% stenosis in the left circumflex artery the sensitivity was as low as 0.69 (95% CI, 0.54–0.81).

## 1 Data

Stress perfusion CMR is increasingly being performed for the noninvasive evaluation of patients with possible CAD (Fihn et al., 2012; Montalescot et al., 2013) [1,2]. Meta-analyses have previously explored the accuracy of stress perfusion CMR to diagnose ischemia-causing lesions at the vessel level (Danad et al., 2017; Dai et al., 2016; Jiang et al., 2016; Takx et al., 2015; Li et al., 2015; Desai and Jha, 2013; Jaarsma et al. 2012; Hamon et al., 2010; Nandalur et al. 2007) [3-11]. However, they included in the same analysis studies with different definitions of significant CAD (i.e. FFR < 0.75 and < 0.80 or coronary stenosis ≥ 50% and ≥ 70%), magnetic field strength (1.5 or 3 T), and study protocol (integration or not of LGE into stress perfusion protocol). Furthermore, they included studies with semi-quantitative assessment although it is rarely used in the clinical setting. In this article, we present pooled data of 34 studies (6091 arteries) with the aim of expanding knowledge about the accuracy of qualitative stress perfusion CMR for the diagnosis of CAD at the vessel level.

**Fig. 1:**
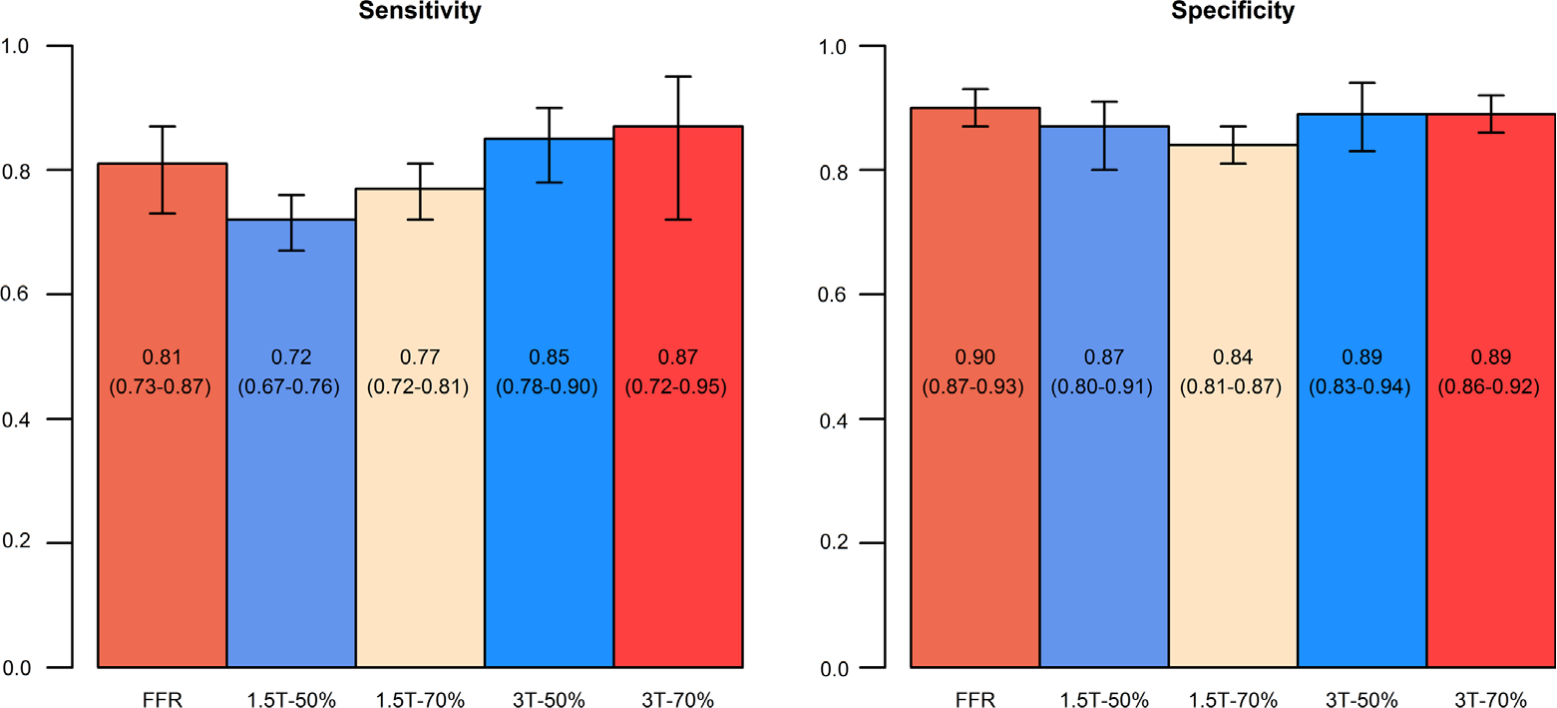
Summary measures of sensitivity and specificity and their 95% confidence intervals for qualitative stress perfusion cardiac magnetic resonance at the vessel level compared with FFR, at 1.5-T for detecting coronary stenosis ≥ 50%, at 1.5-T for detecting coronary stenosis ≥ 70%, at 3-T for detecting coronary stenosis ≥ 50% and at 3-T for detecting coronary stenosis ≥ 70%. FFR, fractional flow reserve; T, Tesla.

Considering only data at the vessel level, analysis of studies using FFR as the reference standard demonstrated a mean sensitivity of 0.81 (95% CI, 0.73–0.87) and a mean specificity of 0.90 (95% CI, 0.87–0.93). Analyses for detecting coronary stenosis ≥ 50% and coronary stenosis ≥ 70% at 1.5-T and for detecting coronary stenosis ≥ 50% and coronary stenosis ≥ 70%, at 3-T, demonstrated a mean sensitivity of 0.72 (95% CI, 0.67–0.76), 0.77 (95% CI, 0.72–0.81), 0.85 (95% CI, 0.78–0.90), and 0.87 (95% CI, 0.72–0.95), respectively; with a mean specificity of 0.87 (95% CI, 0.80–0.91), 0.84 (95% CI, 0.81–0.87), 0.89 (95% CI, 0.83–0.94), and 0.89 (95% CI, 0.86–0.92) (Fig. 1). The results of our analyses are presented in Table 1, Table 2, Table 3.

**Table 1:** Diagnostic performance of qualitative stress perfusion CMR against FFR at the vessel level.

	Studies	Vessels	Sensitivity	Specificity	LR+	LR−	DOR	I^2^	AUC
	(n)	(N)	(95% CI)	(95% CI)	(95% CI)	(95% CI)	(95% CI)		
Overall	9	1689	0.81	0.90	8.64	0.22	44	0%	0.928
			(0.73–0.87)	(0.87–0.93)	(5.69–12.50)	(0.15–0.31)	(22–91)		
< 0.75	5	1071	0.80	0.92	9.91	0.22	46	0%	0.917
			(0.68–0.89)	(0.88–0.94)	(5.82–15.50)	(0.12–0.36)	(16–127)		
< 0.80	5	927	0.82	0.90	8.20	0.21	46	3.9%	0.939
			(0.75–0.87)	(0.82–0.94)	(4.40–14.30)	(0.15–0.29)	(20–103)		
1.5 T	3	732	0.81	0.91	9.68	0.23	43	2.9%	0.902
			(0.58–0.93)	(0.84–0.95)	(3.78–19.20)	(0.08–0.49)	(8–232)		
3 T	6	957	0.81	0.90	8.47	0.21	45	0%	0.934
			(0.74–0.86)	(0.84–0.94)	(4.90–13.80)	(0.15–0.29)	(22–94)		
Perfusion	5	1008	0.83	0.91	9.94	0.19	52	5.7%	0.943
			(0.75–0.89)	(0.87–0.94)	(5.97–15.60)	(0.12–0.28)	(23–120)		
Perfusion/LGE	5	990	0.82	0.90	8.06	0.22	43	0%	0.920
			(0.69–0.90)	(0.84–0.93)	(4.58–13.20)	(0.11–0.36)	(14–105)		

CMR, cardiac magnetic resonance; FFR, fractional flow reserve; CI, confidence interval; LR+, positive likelihood ratio; LR−, negative likelihood ratio; DOR, diagnostic odds ratio; AUC, area under summary receiver-operating characteristic curve; LGE, late gadolinium enhancement.

**Table 2:** Diagnostic performance of qualitative stress perfusion CMR at 1.5 T against coronary angiography at the vessel level.

	Studies	Vessels	Sensitivity	Specificity	LR+	LR−	DOR	I^2^	AUC
	(n)	(N)	(95% CI)	(95% CI)	(95% CI)	(95% CI)	(95% CI)		
**≥ 50% stenosis**									
Overall	11	1970	0.72	0.87	5.49	0.33	18	0%	0.871
			(0.67–0.76)	(0.80–0.91)	(3.71–7.94)	(0.28–0.38)	(11–30)		
LAD	7	458	0.79	0.86	6.17	0.24	26	0%	0.936
			(0.73–0.85)	(0.75–0.93)	(3.18–11.30)	(0.18–0.32)	(12–58)		
LCx	7	468	0.69	0.88	6.09	0.36	18	0%	0.881
			(0.54–0.81)	(0.76–0.94)	(3.09–11.30)	(0.23–0.51)	(9–37)		
RCA	7	465	0.77	0.86	6.04	0.28	25	5.5%	0.898
			(0.64–0.86)	(0.73–0.94)	(2.98–11.50)	(0.17–0.41)	(11–59)		
Perfusion	7	1391	0.73	0.87	5.83	0.31	21	0%	0.883
			(0.68–0.78)	(0.78–0.93)	(3.45–9.48)	(0.27–0.37)	(11–39)		
Perfusion/LGE	7	1011	0.72	0.85	4.92	0.33	15	3.1%	0.864
			(0.65–0.78)	(0.79–0.90)	(3.48–6.83)	(0.26–0.42)	(9–25)		
				
**≥ 70% stenosis**									
Overall	13	2710	0.77	0.84	4.91	0.27	19	6.0%	0.885
			(0.72–0.81)	(0.81–0.87)	(4.01–5.99)	(0.22–0.33)	(13–27)		
LAD	8	650	0.82	0.82	4.48	0.23	21	0%	0.920
			(0.74–0.87)	(0.76–0.86)	(3.33–5.93)	(0.16–0.32)	(11–39)		
LCx	8	669	0.74	0.85	5.06	0.31	17	0%	0.878
			(0.66–0.81)	(0.79–0.90)	(3.56–7.07)	(0.23–0.40)	(11–27)		
RCA	9	717	0.78	0.86	5.46	0.27	21	0%	0.906
			(0.70–0.84)	(0.81–0.89)	(4.09–7.21)	(0.19–0.35)	(14–33)		
Perfusion	10	2362	0.78	0.83	4.64	0.27	19	15.1%	0.886
			(0.72–0.83)	(0.79–0.86)	(3.73–5.72)	(0.21–0.34)	(12–28)		
Perfusion/LGE	9	1455	0.78	0.87	6.15	0.25	24	0%	0.906
			(0.74–0.82)	(0.84–0.89)	(4.94–7.59)	(0.20–0.31)	(17–36)		

CMR, cardiac magnetic resonance; CI, confidence interval; LR+, positive likelihood ratio; LR−, negative likelihood ratio; DOR, diagnostic odds ratio; AUC, area under summary receiver-operating characteristic curve; LAD, left anterior descending; LCx, left circumflex; RCA, right coronary artery; LGE, late gadolinium enhancement.

**Table 3:** Diagnostic performance of qualitative stress perfusion CMR at 3 T against coronary angiography at the vessel level.

	Studies	Vessels	Sensitivity	Specificity	LR+	LR−	DOR	I^2^	AUC
	(n)	(N)	(95% CI)	(95% CI)	(95% CI)	(95% CI)	(95% CI)		
**≥ 50% stenosis**									
Overall	5	978	0.85	0.89	8.38	0.17	60	22.6%	0.942
			(0.78–0.90)	(0.83–0.94)	(4.95–13.50)	(0.11–0.26)	(23–154)		
Perfusion	1	–	–	–	–	–	–	–	–
Perfusion/LGE	4	795	0.85	0.89	8.22	0.18	57	32.5%	0.938
			(0.74–0.92)	(0.80–0.94)	(4.23–14.80)	(0.09–0.30)	(18–181)		
				
**≥ 70% stenosis**									
Overall	4	669	0.87	0.89	8.08	0.16	55	0.1%	0.941
			(0.72–0.95)	(0.86–0.92)	(5.69–10.90)	(0.06–0.32)	(20–156)		
Perfusion	1	–	–	–	–	–	–	–	–
Perfusion/LGE	3	489	0.90	0.90	9.18	0.12	83	0%	0.949
			(0.74–0.97)	(0.86–0.93)	(6.50–12.60)	(0.04–0.29)	(28–250)		

CMR, cardiac magnetic resonance; CI, confidence interval; LR+, positive likelihood ratio; LR-, negative likelihood ratio; DOR, diagnostic odds ratio; AUC, area under summary receiver-operating characteristic curve; LGE, late gadolinium enhancement.

## 2 Experimental design, materials and methods

### 2.1 Data sources and searches

A systematic review and meta-analysis was performed following Preferred Reporting Items for Systematic reviews and Meta-Analyses (PRISMA) criteria [47].

Papers were retrieved in Pubmed, Web of Science and the Cochrane Library from inception to 10 September 2017. No search restrictions were applied [12], [13], [14], [15], [16], [17], [18], [19], [20], [21], [22], [23], [24], [25], [26], [27], [28], [29], [30], [31], [32], [33], [34], [35], [36], [37], [38], [39], [40], [41], [42], [43], [44], [45].

### 2.2 Selection criteria

Detailed description of selection criteria of the papers is described elsewhere [46]. In particular, we focused on studies using qualitative stress perfusion CMR for the diagnosis of CAD compared to ICA or FFR at the vessel level.

### 2.3 Data synthesis

Among studies using ICA as the reference standard, we performed subgroup analyses according to magnet strength (1.5-T or 3-T) and the threshold used to define significant CAD (≥ 50% or ≥ 70%). We also performed a separate analysis for studies using FFR as the reference standard. When feasible, we performed further subgroup analysis according to integration or not of LGE into stress perfusion protocol and the coronary artery (left anterior descending, left circumflex and right coronary artery).

### 2.4 Statistical analysis

Summary statistics (sensitivity, specificity, likelihood ratios) were estimated using bivariate models and random effect approaches, from the summary receiver operating characteristic (SROC) curve [48]. We calculated the diagnostic odds ratio (DOR) using a DerSimonian-Laird random-model and the AUC (area under SROC curve) using Holling's proportional hazards model. Statistical heterogeneity was assessed with the I^2^ statistic [49]. Analyses were performed using the software R version 3.4.1 combined with the package ‘mada’ (meta-analysis of diagnostic accuracy) [50], [51].
