# Supplementary material for: Data on diagnostic performance of stress perfusion cardiac magnetic resonance for coronary artery disease detection at the vessel level
Source: Data Brief. 2017 Dec 7;16:869–75. doi: 10.1016/j.dib.2017.11.096 (PMC5847623; doi:10.1016/j.dib.2017.11.096)
Supplement: Supplementary file 1 — Supplementary material [file mmc1.docx]

**Declarations of interest**

None.
